# 14^th^ century *Yersinia pestis* genomes support emergence of *pestis secunda* within Europe

**DOI:** 10.1371/journal.ppat.1011404

**Published:** 2023-07-18

**Authors:** Cody E. Parker, Alina N. Hiss, Maria A. Spyrou, Gunnar U. Neumann, Philip Slavin, Elizabeth A. Nelson, Sarah Nagel, Xandra Dalidowski, Susanne Friederich, Johannes Krause, Alexander Herbig, Wolfgang Haak, Kirsten I. Bos

**Affiliations:** 1 Max Planck Institute for the Science of Human History, Jena, Germany; 2 Max Planck Institute for Evolutionary Anthropology, Leipzig, Germany; 3 Institute for Achaeological Sciences, University of Tübingen, Tübingen, Germany; 4 Division of History, Heritage and Politics, University of Stirling, Stirling, Scotland, United Kingdom; 5 Landesamt für Denkmalpflege und Archäologie, Sachsen-Anhalt, Halle (Saale), Germany; Institut Pasteur of Shanghai Chinese Academy of Sciences, CHINA

## Abstract

*Pestis secunda* (1356–1366 CE) is the first of a series of plague outbreaks in Europe that followed the Black Death (1346–1353 CE). Collectively this period is called the Second Pandemic. From a genomic perspective, the majority of post-Black Death strains of *Yersinia pestis* thus far identified in Europe display diversity accumulated over a period of centuries that form a terminal sub-branch of the *Y*. *pestis* phylogeny. It has been debated if these strains arose from local evolution of *Y*. *pestis* or if the disease was repeatedly reintroduced from an external source. Plague lineages descended from the *pestis secunda*, however, are thought to have persisted in non-human reservoirs outside Europe, where they eventually gave rise to the Third Pandemic (19^th^ and 20^th^ centuries). Resolution of competing hypotheses on the origins of the many post-Black Death outbreaks has been hindered in part by the low representation of *Y*. *pestis* genomes in archaeological specimens, especially for the *pestis secunda*. Here we report on five individuals from Germany that were infected with lineages of plague associated with the *pestis secunda*. For the two genomes of high coverage, one groups within the known diversity of genotypes associated with the *pestis secunda*, while the second carries an ancestral genotype that places it earlier. Through consideration of historical sources that explore first documentation of the pandemic in today’s Central Germany, we argue that these data provide robust evidence to support a post-Black Death evolution of the pathogen within Europe rather than a re-introduction from outside. Additionally, we demonstrate retrievability of *Y*. *pestis* DNA in post-cranial remains and highlight the importance of hypothesis-free pathogen screening approaches in evaluations of archaeological samples.

## Author summary

We present two high-coverage and three low-coverage ancient *Y*. *pestis* genomes that represent previously described diversity within a narrow time interval that immediately post-dates the Black Death (the *pestis secunda*), and a newly identified precursor strain. Viewed in context with recent historical revelations that define the time-span between the end of the Black Death and the emergence of the *pestis secunda* to a three-year interval, our analysis strongly supports this outbreak, similar to other post-Black Death outbreaks, as having originated from a local source of *Y*. *pestis* diversification.

## Introduction

*Yersinia pestis*, the bacterium responsible for plague [1], is primarily spread from rodents to humans and other mammals via infected fleas [2–4]. At least three historically documented plague pandemics are known, and involvement of *Y*. *pestis* in each has been confirmed through DNA analysis: the First Pandemic, which began with the Justinianic plague/Plague of Justinian (6^th^ to 8^th^ century CE) [5–7], the Second Pandemic, which started with the Black Death (mid-14^th^ to early 19^th^ century CE) [8–10], and the modern Third Pandemic (mid-19^th^ and 20^th^ centuries) [11]. Recent evidence has shown that *Y*. *pestis* infection occurred as early as ~5,200 years ago [12,13], although the health impact of these cases on prehistoric societies remains a subject of scholarly debate.

From a genomic perspective, the most widely studied of the two historical *Y*. *pestis* scourges is the Second Pandemic, which includes the Black Death (1346-1353CE) [10] and the subsequent waves of resurgent outbreaks that followed for several hundred years [14]. The Black Death is thought to have started with the introduction of a single, clonal strain of *Y*. *pestis* that has been recently identified in Central Asia [15]. After spreading to the Black Sea region, it was carried via trade [10,16] through Constantinople before entering Europe via the Italian city of Messina [17,18], all in 1347. The Black Death then spread rapidly throughout most of Europe before abating in 1353 CE. Shortly thereafter, waves of infection periodically resurfaced in Europe until the early 19^th^ century CE, each resulting in episodes of high mortality [19–21].

Chronologically, the first known resurgence of plague in Europe after the Black Death, referred to as the *pestis secunda* [22], has been genetically observed in remains excavated from plague-associated burials in Bergen Op Zoom, the Netherlands (archaeologically dated to post-1358 CE, and to 1359–60 CE through historic context) [8,23], from the London St. Mary’s Graces plague cemetery (historically dated to 1361 CE) [9,24] and from a cemetery in Bolgar, Russia (post 1362 CE, though possibly 1363–4 CE based on historical context) [25]. The strains detected in these *pestis secunda* burials all differ from that responsible for the Black Death by only two diagnostic chromosomal single-nucleotide polymorphisms (SNPs): one at nucleotide position 699,494 following annotation of the CO92 AL590842 reference genome (p3, a synonymous G to A transition in the *rpoD* gene) and another at position 2,262,577 (p4, a non-synonymous G to T transversion affecting a hypothetical protein whose function remains unclear) [8,9,14,23,25]. Additionally, the *pestis secunda* strains thus far identified do not group phylogenetically within the greater diversity reported for other post-Black Death mortality events throughout Europe, which are argued to have evolved locally [14,25]. Instead, genomic evidence indicates that the known lineages common to this cluster extended eastward beyond Europe into Asia and subsequently Africa, and later gave rise to the prolific *Y*. *pestis* branch 1, which is highly diverse and is associated with the globally distributed Third Pandemic [11,22].

The locations of plague foci that could have seeded the various post-Black Death *Y*. *pestis* resurgences are debated. Recent genetic studies of historical *Y*. *pestis* strains recovered from across Eurasia in the medieval and post-medieval periods have revealed a pattern of diversification with the Black Death genotype at the root [14]. This strongly supports the 14^th^ century pandemic event as the ultimate source of Second and Third Pandemic outbreaks in humans, with accumulated diversity in the Second Pandemic resulting from continued local evolution within Europe [14,25]. From an ecological perspective, the waves of post-Black Death infection appear to be correlated with factors associated with the rise and decline of regional sylvatic rodent populations [26,27]: As rodent populations expand and reach urban centres, susceptible commensal rodents such as the black rat (*Rattus rattus*) can become infected, which can lead to epidemics in humans [28,29]. In the context of the Second Pandemic, the frequency of outbreaks is consistent with *Y*. *pestis* persistence in local rodents where the bacteria periodically receded, evolved, and re-emerged as their populations fluctuated [26,30].

South-Central Germany has recently been proposed as a plausible candidate location where plague may have become endemic in a local rodent population in the course of the Black Death, which eventually led to a spill-over event that gave rise to the *pestis secunda* [30–32]. As historical records first document waves of infection around major population centres and along corresponding trade routes, an alternative hypothesis posits that subsequent waves of infection are better explained by a series of continuous re-introductions of plague from either the original source of the Black Death or elsewhere in Asia [23,33–35]. It should be noted, however, that the vast majority of all late medieval plague data is recovered from urbanized/commercialized areas rather than from more sparsely populated rural areas: Hypotheses regarding the locations of epidemic emergences could thus be reflective of inherent biases in the availability of historic records rather than actual epidemiological phenomena. Additionally, involvement of local rodents is underexplored as genetic evidence of *Y*. *pestis* infection in medieval rodent populations has only recently been observed [36]. From a genetic standpoint, observational bias results from a dominant focus on skeletal collections for which a plague outbreak is either historically documented or is suspected based on burial context. These are often from urban centres, where rescue excavations are common. So-called *Wüstungen* (German for abandoned settlements) and their associated graveyards are common in the Medieval Period, but have thus far been underutilized in genomic investigations, despite their potential connections to demographic collapse related to infectious disease. Together these phenomena have the potential to increase the resolution of both an outbreak’s origin and the context of its re-emergence.

A more systematic survey of *Y*. *pestis* DNA recovery from a wide range of spatio-temporal contexts both within and outside Europe has the potential to clarify the geographic origins of these recurrences [14,23,33]. Genetic data representative of the *pestis secunda*, for example, is currently limited to only three sites: one in each of The Netherlands [8,23], London [9], and the Volga region of Russia [25]. Importantly each genome shares two derived positions that distinguish it from the Black Death; none display the transitional state between the two pandemics that would reveal key aspects of the *pestis secunda*’s spatio-temporal emergence. Here, through a hypothesis-free approach to pathogen screening of human remains from burials in medieval Germany that lack the typical contexts associated with epidemic events (in terms of both demography and historical context), we present five *Y*. *pestis* genomes that further explore the genetic links between the Black Death and the *pestis secunda*. These data parsimoniously support precipitation of the *pestis secunda* as a local phenomenon rather than a disease reintroduction from afar.

## Results

**Fig 1:**
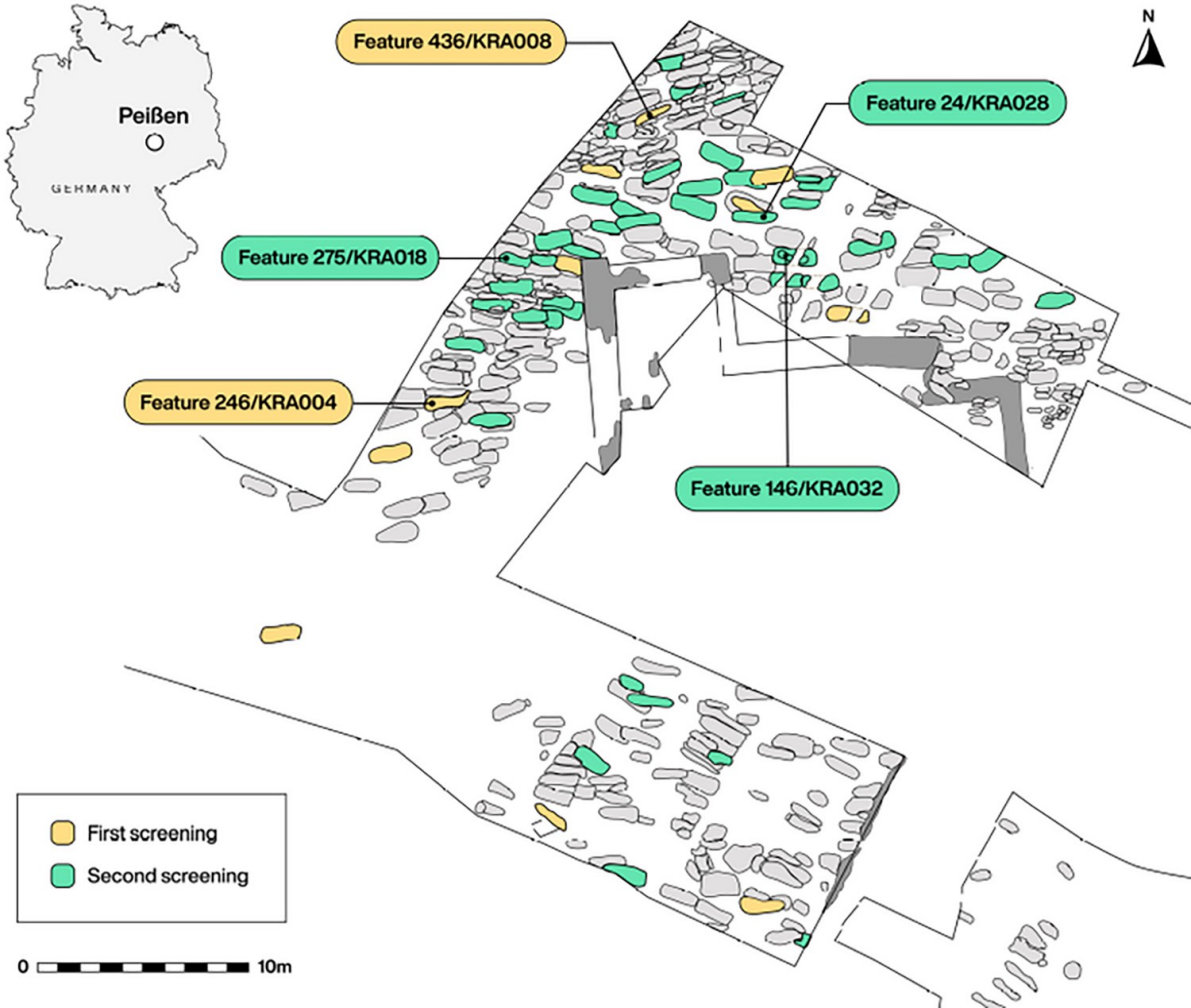
Map of the cemetery excavation site at Krakauer Berg, near Halle (Saale), Saxony-Anhalt, Germany. Graves highlighted in yellow mark those initially screened, while the green highlighted features represent those utilized for the second, targeted *Y*. *pestis* screening effort. Labelled graves denote individuals where *Y*. *pestis* has been detected. Figure generated by Michelle O’Reilly. The map was created using QGIS [1]https://qgis.org/en/site/ and uses Natural Earth vector map data from [2]https://www.naturalearthdata.com/downloads/.

Here we present the rare occasion to conduct pathogen screening of individuals recovered from an abandoned European settlement, the 14^th^ century Krakauer Berg cemetery in Halle (Saale), Germany (Fig 1). The reasons behind its abandonment are unknown, and details on the infectious disease experience of its former inhabitants, especially with highly lethal pathogens such as *Y*. *pestis*, may provide some relevant context to inform on its history. In a separate study, 247 single-stranded DNA libraries from eleven individuals were investigated for comparative ancient human DNA recovery based on bulk DNA (shotgun) content across various skeletal elements [37]. A non-targeted screening of these extensive data for ancient pathogen DNA using the HOPS pipeline [38,39] revealed an expected constellation of oral bacteria in dental samples, as well as confirmation of hepatitis B virus infection in two individuals as previously reported [40]. Further to these findings, this process resulted in the discovery of two additional individuals (KRA004 and KRA008) who showed possible genetic evidence of *Y*. *pestis* infection. Subsequent mapping to the CO92 reference genome (accession number: AL590842.1) provided further evidence for the preservation of *Y*. *pestis* DNA in eight libraries stemming from separate sampling locations within each of these individuals (S1–S3 Figs; S1 Table). Based on the number of uniquely mapped reads per million total sequenced reads, these included cementum from individuals KRA004 and KRA008 (4.79 and 1.41 unique reads/M, respectively), dentin (8.76 and 1.45 unique reads/M, respectively), and pulp chamber (217 and 4.78 unique reads/M, respectively) from both individuals, as well as the superior vertebral arch (3.00 unique reads) and femur (3.02 unique reads) from KRA004 (S1 Table). While these results lend credence to previous assumptions that sampling from the teeth, specifically in the region of the dental pulp chamber, is an optimal strategy for recovery of blood-borne pathogens such as *Y*. *pestis* [41], we also demonstrate detectable preservation of pathogen DNA in post-cranial remains.

Given the demonstrated success of the dental sampling locations, 50 teeth from an additional 43 individuals excavated from the site were obtained to further explore plague’s impact on the mortality of the individuals buried in the Krakauer Berg cemetery. Pulverised material from the dental pulp chambers was screened for the presence of *Y*. *pestis* DNA using a targeted PCR assay for the *pla* region of the pPCP1 plasmid [42]. Amplification products were observed in three of these individuals, namely KRA018, KRA028, and KRA032 (S2 Table and S4 Fig). Double-stranded DNA libraries prepared using a partial uracil-DNA glycosylase (UDG) treatment were then generated from each of the candidate extracts [43]. Together this yielded 11 libraries (eight single-stranded and three double-stranded) from the five putative plague victims for further analyses. An additional double-stranded library with full UDG treatment was generated from the dental pulp chamber extract of KRA004 to verify SNP authenticity against the single-stranded data, which were not treated with UDG and therefore retained a greater proportion of damaged template molecules.

All candidate libraries from the five individuals were enriched for *Y*. *pestis* DNA using in-solution capture [44], and were further sequenced (2x75-cycle chemistry on the Illumina platform) to a depth of ca. 10 million reads each. As initial analyses of individuals KRA028 and KRA032 resulted in genomes of low coverage (< 1-fold, S3 Table), a second double-stranded partial UDG treated library, as well as a further non-UDG treated single-stranded DNA library, were generated from the corresponding extracts, which were later enriched and sequenced as described above. Reads mapping to the *Y*. *pestis* reference genome were authenticated as ancient based on accord between patterns of deamination observed for both *Y*. *pestis* and human reference genome mapping (GRCh37[hg19]); accession number: GCF_000001405.13, S5 Fig, S3 and S4 Tables).

**Fig 2:**
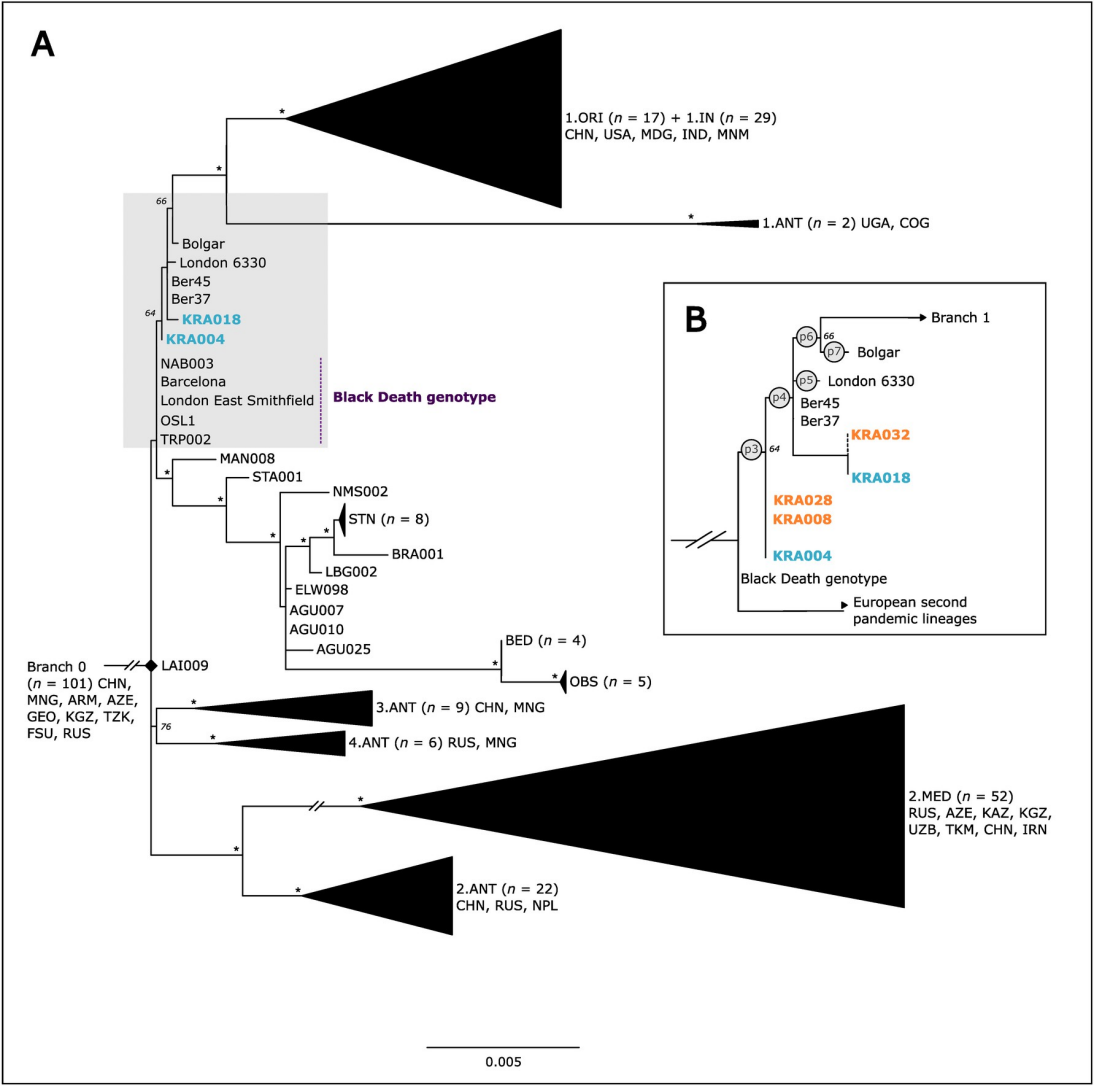
**(A)** Historical *Y*. *pestis* diversity including the Second, and Third Pandemics. New high-coverage genomes from Krakauer Berg are denoted in blue. Strain and lineage acronyms follow conventions established in the published literature [5,14,65]. Bootstrap values above 90% are denoted with an asterisk, and numeric values of all lower percentages are shown. **(B)** Zoomed view of the *Y*. *pestis* diversity within *pestis secunda* (grey area in A). High coverage genomes are denoted in blue, whereas the tentative, manual placements of low-coverage genomes are denoted in orange. Lineage-characteristic SNPs (Table 1) are indicated in circles at their respective branches.

Of these twelve libraries, *Y*. *pestis* genomes with a mean coverage of 10-fold or more were reconstructed from both the single- and double-stranded libraries from the dental pulp chamber of KRA004 (ca. 40-fold and 11-fold coverage, respectively, for a combined 51-fold genome) as well as the double-stranded library from this sampling location of KRA018 (ca. 20-fold, S3 Table). Datasets for the enriched products of KRA008, KRA028, KRA032 yielded average genomic coverages of 0.7-fold, 3.3-fold, and 0.5-fold respectively, and hence were considered too low for inclusion in downstream analyses (S3 Table). Single nucleotide polymorphisms (SNPs) were called at a minimum of 3-fold coverage and 90% of reads supporting the SNP allele (Tables 1 and S5). To assess the placement of the recovered *Y*. *pestis* strains within the greater diversity of historical *Y*. *pestis*, a bootstrapped maximum likelihood tree was constructed using RAxML [45] (98% partial deletion, 1000 iterations) for the combined enriched libraries from all sampling locations (cementum, dentin, dental pulp, superior vertebral arch, and femur) from individual KRA004 and the enriched dental pulp library from individual KRA018 (Fig 2A).

**Table 1:** SNP table showing the seven diagnostic Second Pandemic *Y*. *pestis* SNPs (p1-p7 [25]) and additional *pestis secunda* diversity comparing profiles of the Black Death genotype, all Krakauer Berg genomes (KRA0—), known examples of *pestis secunda* (Bergen op Zoom, London St. Mary’s Graces, Bolgar), and the modern CO92 reference (accession number: AL590842). Amongst the two high-coverage genomes (blue), a minimum of 3 reads covering each position were required for SNP identification, while SNPs in the low-coverage genomes (orange) were evaluated at minimum 1-fold coverage, with number of reads supporting the SNP shown in parentheses. Sites where this coverage was not achieved (for both high or low-coverage genomes) are denoted with Ns. Nucleotides diagnostic of *pestis secunda*/Branch 1 strains are shown in bold.

	SNP (Position in reference to the CO92 reference genome)								
Individual/Site	189,227 (p1)	1,871,476 (p2)	699,494 (p3)	2,262,577 (p4)	4,301,295 (p5)	3,806,677 (p6)	3,643,387 (p7)	1,009,185	1,526,622
**Black Death**	C	G	G	G	G	T	G	C	T
**KRA004**	C	G	**A**	G	G	T	G	C	T
**KRA008**	N (0)	N (0)	**A** (1)	G (2)	N (0)	N (0)	G	N (0)	N (0)
**KRA018**	C	G	**A**	**T**	G	T	G	T	C
**KRA028**	C	G	**A** (3)	G (2)	G	T	G	C (5)	T (2)
**KRA032**	N (0)	N (0)	**A** (4)	N	G	N (0)	N (0)	T (1)	N (0)
**Bergen-op-Zoom**	C	G	**A**	**T**	G	T	G	C	T
**London, St. Mary Graces**	C	G	**A**	**T**	T	T	G	C	T
**Bolgar**	C	G	**A**	**T**	G	**C**	T	C	T
**CO92**	C	G	**A**	**T**	G	**C**	G	C	T

All genomes were investigated with regard to their SNP profiles, with special attention given to status at positions p3 and p4 that have previously defined the *pestis secunda* lineages [25]. Our manual evaluation of the partial SNP profiles for the lower coverage genomes (KRA008, KRA028, and KRA032) were also used to extrapolate their placement in the phylogenetic tree (2B, S6 Figs). All genomes presented here carry the derived state at position p3, thus confirming their phylogenetic grouping within *pestis secunda* as inferred through the maximum likelihood approximation. Importantly, position p4 did not yield consistent allele calls across the dataset: While present in the derived state for KRA018, the KRA004 genome carries the ancestral state, i.e. the nucleotide call is identical to the Black Death genotype. Although coverage at this position was too low for genotype assessment in the data for KRA028 and KRA008 (coverage was <3 reads), the ancestral state for p4 was detected by visual inspection of the alignment (2B, S6B Figs). The low coverage of KRA032 precluded further assessment at this position (S6B Fig).

In addition, two derived non-homoplastic positions unique to KRA018 were detected [positions 1,009,185 (C → T) and 1,526,622 (T → C) with respect to the CO92 reference genome (S7 Fig and S5 Table) at 26-fold and 4-fold coverage respectively], indicating further divergence of this genome from the others presented here. Given that the variant SNP at position 1,009,185 was also found in KRA032 [(S6C Fig), 1-fold coverage], we can infer that KRA032 is likely to cluster with KRA018. Functional analysis of variant positions was performed using SnpEff [46] and analysis revealed the SNP at position 1,009,185 to represent a non-synonymous glutamine to arginine mutation (S6 Table). The gene affected by the mutation (YMO0919) likely codes for a protein, though with unknown function. The other SNP at position 1,526,622 is located in an intergenic region. A presence/absence analysis of both high coverage genomes targeting genes known to be involved in *Y*. *pestis* virulence indicates that, aside from a filamentous prophage acquired by 1.ORI (to which CO92 belongs), all expected virulence genes were present with no additional gene loss detected (S8 Fig).

Radiocarbon dates were available for KRA004 (1284–1392 CE, Cal 2-sigma), and KRA008 (1301–1402 CE, Cal 2-sigma) from a previous publication [37], and additional dating was performed for KRA018 (1283–1389 CE, Cal 2-sigma).

## Discussion

Although *Y*. *pestis* is among the most densely studied ancient pathogens to date, many questions persist regarding its evolutionary history and past distribution. Here, through the application of a non-targeted screening approach, we demonstrate the unexpected presence of *Y*. *pestis* in skeletal material interred in the absence of typical epidemic-related contexts, in contrast to nearly all previous examples of historic *Y*. *pestis* findings. Two of the genomes presented here, namely those from individuals KRA018 and KRA032, group within the diversity previously documented for plague burials linked to the *pestis secunda*. Three additional genomes (KRA004, KRA008, and KRA028), however, are clearly basal to the diversity that previously defined the *pestis secunda* cluster (Fig 2).

Our observation that the five genomes from the same cemetery represent two *Y*. *pestis* phylogenetic clusters could reflect two possible scenarios: local evolution in the living community (human or rodent), or reintroduction. Genomes with a derived p3 and ancestral p4 SNP profile reflect an earlier stage of plague outbreak, and those with the derived state in both p3 and p4 are associated with later stages of the pandemic. Here our p4 cluster (KRA018 and KRA032) includes two additional private mutations, thus making it three nucleotide differences from the genotype of the putative *pestis secunda* onset. Historical evidence suggests that *pestis secunda* outbreaks lasted, on average, half a year, which is likely too short a time interval for acquisition of three polymorphisms during an outbreak when viewed in the context of genomic data from other single spatio-temporal Second Pandemic events such as the Black Death [9,25], Stans (c.1485-1635 CE) [14], Marseille (1720–1722 CE) [24] and London, Bedlam (17^th^ century) [14], which show near clonality in their strains. The possibility that genomes KRA032 and KRA018 reflect a resurgence of the pandemic a few years later is in better accord with the current data: as both derive from the *Y*. *pestis* genotype that predates acquisition of SNPs p5 and p6 (in London and Bolgar, respectively), they are reflective of independent and parallel diversity that accumulated at some point in Europe during the decade-long *pestis secunda* [32]. Halle was an important salt producing and trading centre, with commercial ties to other parts of the German Empire and beyond [47–49]. The presence of the p4 SNP in the Netherlands and England indicates that this strain achieved a wide geographic distribution. Our level of genetic resolution also suggests that the strains identified here were all of similar virulence potential to those associated with the Black Death, which were transmitted across vast distances in short intervals of time.

While geographical ranges of the *Y*. *pestis* genotypes presented here are unknown, recovery from the Krakauer Berg cemetery of the most basal lineage in the *pestis secunda* cluster identified thus far could be viewed as support for the hypothesis that the pandemic emerged regionally, or somewhere within the regional network of established trade contacts [30]. This in turn supports historical interpretation of plague’s endemic status in Europe in the 1350s CE, with the first human cases of the *pestis secunda* reported in 1356 CE [30], just three years after conclusion of the Black Death.

Initial estimates for the onset of the *pestis secunda* suggest it occurred sometime between 1353 CE (the end of the Black Death in Europe) and 1359–1360 CE, the inferred historical date of the Bergen op Zoom burials which comprise the first reported *Y*. *pestis* genomes confirmed to represent this widespread mortality event [8,23]. The outbreak reached England only a few years later (commencing in London in spring 1361 CE) and necessitated the consecration of the London St. Mary Graces cemetery in the same year for victims of the pandemic. While establishment of a precise geographic origin of the pandemic goes beyond our reach, details of the pandemic’s early development can be refined using recently uncovered historical data, including chronicles, probated wills and local authority records that together indicate first historical evidence of a potential *pestis secunda* wave in summer 1356 CE in today’s South-Central Germany. Data from historical sources presented elsewhere [30] form a narrative that describes the course of plague’s spread in the currently-defined Germanic lands and beyond. In the following year (1357 CE) plague spread out of Hesse: in the west it reached Westphalia and east Rhineland-Palatinate; in the east, it appeared in Thuringia and Saxony-Anhalt; in the north, it was circulating in the western parts of North Rhine and southern parts of Lower Saxony, while in the south, it proliferated in the eastern part of Baden-Württemberg and Bavaria, eventually reaching Lake Constance. From there the pandemic extended into north-west Germany, the eastern part of the bishopric of Constance (including the north-eastern tip of Switzerland), and the hinterland of Strasbourg. By 1359 CE it spread further to the Netherlands in the north-west and Pomerania in the north-east, Austria in the south-east, and the Zurich hinterland and Alsace in the south-west. While no surviving textual records describe the *pestis secunda* outbreak in Halle/Krakauer Berg or its immediate hinterland, plague was reported in both Meiningen (20km south-west of Halle) and Magdeburg (90km north of Halle) in 1357 CE, implying that Halle (Saale), situated between the two cities, experienced its first outbreak most likely in the same year [50,51]. Drawing upon the synthesis of these historical data we propose a temporal placement of the burials KRA004, KRA008, and KRA028 within a narrow time interval of 1356–1357 CE. Burials from the later interval, KRA018 and KRA032, likely date from 1366 CE at the latest, which corresponds to the conclusion of this pandemic wave in Europe [32]. The above scenario is consistent with calibrated radiocarbon dates for the individuals of this study (KRA004: 1284–1392 CE, calibrated 2-sigma range [37], KRA008: 1301–1402 CE, cal. 2-sigma [36], and KRA018: 1283–1389 CE, cal. 2-sigma). These data also indicate an average acquisition of one SNP in the circulating bacterial population approximately every three to four years during this outbreak for the genomic regions considered here, which is consistent with inferred mutation rates for branch 1 of *Y*. *pestis* [14].

The basal phylogenetic position of genomes KRA004, KRA008 and KRA028 (derived p3) and the progressive accumulation of derived positions in the genomes from Bergen-op-Zoom and London in the West (derived p4 and p5, respectively), and later in Bolgar on the Volga-Kama confluence in the East (derived p6) pairs well with historical evidence of *pestis secunda* spread in West Eurasia and its eventual seeding of diverse lineages in China associated with the prolific Third, or modern, Pandemic [10,52]. The *pestis secunda* reached the Netherlands in 1359–60 CE, continued onto London in March 1361 CE (from Gascony or from the Low Countries) and circulated in the Volga region in 1363–4 CE, having arrived there, most likely, from the Azov Sea littoral, where it is documented in 1362 CE. From there, the plague would continue westwards into Russian principalities (1364–5 CE) and Lithuania (1366 CE), where it finally abated [30]. In the east, plague broke out in Armenia and Georgia in 1364–6 CE [53,54], but its subsequent global proliferation leading to the onset of the Third Plague Pandemic in 1855 CE in Western Yunnan cannot, at this point, be historically reconstructed.

While teeth have been a popularly targeted sampling location for aDNA recovery of *Y*. *pestis* [41,55], our data, even if only based on comparative results obtained from two individuals, further supports their use (specifically the dental pulp chamber) as a preferred sampling location for investigation of pathogens present in the bloodstream at the time of death. We demonstrate, however, the benefit offered by routine screening of postcranial elements, as *Y*. *pestis* infection was also detected in both a vertebra and femur of individual KRA004, albeit with rather low recovery of pathogen DNA when compared to the tooth. This is especially important in cases where it may be possible to perform complementary sampling of teeth from individuals where *Y*. *pestis* infection is detected via molecular analysis of postcranial remains. Our independent identification of two previously reported hepatitis B infections [40] demonstrates accuracy in our hypothesis-free screening approach for pathogens and its application to future identifications of disease from bulk DNA data.

In many cases human remains that are investigated for molecular detection of infectious diseases are screened with a narrow focus on a single target pathogen. Similarly, studies undertaken with a focus on population genetics have, in the past, neglected to screen the recovered molecules for potential pathogens, especially in the absence of historical documentation, burial context, or pathological evaluation suggestive of possible infection. While this can be resolved by the retroactive screening of datasets from past projects in combination with new sampling endeavours [56], it may not always be possible to obtain additional material for subsequent analyses should pathogens be detected. Our findings here reinforce the value of routine screening of all datasets for both host and pathogen DNA (when authorized to do so by the relevant cultural authority) as this presents an inexpensive strategy to maximize the amount of information that can be produced from destructive analyses of archaeological remains.

## Conclusion

Drawing from both genetic and historical data resources, this study offers support for the notion that the *pestis secunda* originated within Europe. Additionally, our approach highlights the benefit of pathogen screening in 1) burials that lack features regarded as typical of epidemic contexts, 2) settlements that are known to have been abandoned after intense plague outbreaks, and 3) skeletal elements that have been processed for other molecular applications. Further pairing of genetic results from both human and faunal remains with relevant historical data will provide an optimal strategy to untangle the web of plague dissemination throughout Europe and its neighbouring continents.

## Material and methods

### Sampling and initial screening

Multiple skeletal elements from 11 individuals excavated from the abandoned medieval cemetery at Krakauer Berg, near Peißen, Saxony-Anhalt, Germany were provided by the State Museum of Prehistory, Halle (Saale), Germany. These elements consisted of the petrous portion of temporal bones, molars, clavicles, ribs, vertebrae, metacarpals, distal phalanges, ischia, femora, and tali from each individual [37]. As the subsequent screening for the presence of potential pathogens in these original 247 samples indicated the presence of *Y*. *pestis* in two of eleven individuals, an additional 50 teeth from 43 more individuals were sampled to investigate the potential recovery of additional plague genomes at the site. Radiocarbon dating of ribs from each of the original 11 individuals and the molar root of KRA018 was carried out at the Curt Engelhorn Centre for Archaeometry in Mannheim, Germany.

For all samples in the initial screening, bone powder was generated from multiple sampling locations on each element in a dedicated ancient DNA (aDNA) clean room facility at the Max Planck Institute for the Science of Human History in Jena, Germany. DNA was extracted from ~50-75mg of bone powder from 23 sampling locations across ten skeletal elements gathered from eleven individuals each using a modified DNA extraction protocol optimized for the recovery of ancient molecules [37,57]. The resulting DNA from those extractions was then used to generate single-stranded shotgun DNA libraries via automation [58], which were then sequenced on an Illumina HiSeq 4000 platform (75bp paired end, ca. 5 million reads) and screened for the presence of *Y*. *pestis* using both the EAGER [59], (S1 Table) and HOPS [38,39] pipelines. Reads mapping to the CO92 reference genome were authenticated as ancient through comparison of the observed deamination patterns obtained via mapping to the bacterial reference with those observed via mapping to the human reference genome hg19 using the Burrows-Wheeler Alignment (BWA) [60] and MapDamage [61] tools contained within the EAGER pipeline (S1 and S4 Tables) [59]. Those libraries determined to contain verifiable traces of historical *Y*. *pestis* were then enriched for the pathogen using in-solution targeted capture [43] based on diversity within *Y*. *pestis* [14] and sequenced using the Illumina platform to a depth of ca. 10 million reads. An additional enriched, double-stranded, full UDG-treated library was generated from the same extraction of pulp-chamber powder for individual KRA004 [62] for use in downstream SNP verification, as most reads mapping to the *Y*. *pestis* reference genome were observed in this sample (S1 Table).

After the presence of historical *Y*. *pestis* was confirmed for the site, an additional 50 teeth were collected from 43 individuals, sampled as directed by previously published methods [63]. DNA was extracted from the pulp chamber following the methods described above. Extractions from these additional samples were then screened for the presence of the bacterium using a qPCR assay targeting the *pla* gene located on the pPCP1 *Y*. *pestis* plasmid [42], (S2 Table). Double-stranded partial UDG treated libraries were then generated from each extract that yielded an amplification product [62], (S2 Table). Both bulk DNA libraries and those enriched for *Y*. *pestis* DNA were sequenced using the Illumina platform as above to a depth of ca. 10 million 75bp paired end reads each. As this sequencing effort yielded genomes of low coverage for KRA028 and KRA032, an additional double-stranded (partial UDG) and single-stranded (non-UDG) library was generated for each individual, and further enriched and sequenced as previously described (S3 Table).

### Analysis

Screening of the shotgun sequenced data was done using the EAGER pipeline [59] to generate alignments to the CO92 reference genome [seed length 16, 0.01 mismatch stringency, mapping quality filter 37 for the initial set of libraries, seed length 16, 0.01 mismatch stringency, mapping quality filter 37 for the subsequent libraries (KRA012-KRA054)] and to verify that the mapped reads showed a damage pattern consistent with ancient DNA. Post-enrichment, all adapter-clipped reads from UDG-half libraries were trimmed by 1bp from both the 5’and 3’ ends using fastx trimmer [https://github.com/agordon/fastx_toolkit] to remove damage and re-mapped with more stringent BWA mapping parameters (seed length 32, 0.1 mapping stringency, mapping quality filter of 37). The same parameters were used for the UDG library of KRA004. Libraries that did not receive UDG treatment were not trimmed to prevent loss of data and were instead re-mapped with lenient parameters (seed length 16, 0.01 mismatch stringency, mapping quality filter 37). For further analyses, the bam files for all libraries of KRA004 were concatenated.

SNP calling was performed using with the Unified Genotyper of GATK [64] using the EMIT_ALL_SITES option. We then used a custom java tool MultiVCFAnalyzer [65], (github.com/alexherbig/MultiVCFAnalyzer) to generate a SNP table for positions with minimum 3-fold coverage (S5 Table). Although the combined datasets for KRA028 yielded an average genomic coverage of >3-fold, it was excluded from further analyses due to its <3-fold coverage at informative sites such as p4. Data were compared against 233 modern and 40 ancient genomes [5,66], with *Y*. *pseudotuberculosis* (IP23953) included as an outgroup. SNPs were filtered via SNPEvaluation as described in [5] to remove polymorphic positions that result from probable non-target DNA sequences that persisted in the enriched dataset. A maximum likelihood tree was generated with RAxML [44] based on the General Time Reversible (GTR) substitution model with 1000 bootstrap replicates through application of a 98% partial deletion filter. Phylogenetic placements of low-coverage genomes were done by visual inspection of genome alignments based on the aforementioned SNP analysis of the datasets stemming from individuals KRA008, KRA028, and KRA032 [67] (S6 Fig). Additionally, SnpEff [46] was used to classify the possible effects of SNPs on any associated genes and/or gene products (S6 Table).

The two high coverage genomes retrieved from KRA004 and KRA018 were investigated for presence or absence of known *Y*. *pestis* virulence factors [68] alongside other published ancient (Bolgar 2370, London 6330, Ber45, NAB003, London ES 8124/8291/11972, Barcelona 3031, OBS137, STN014, Altenerding, RISE509) and modern (CO92, *Y*. *pseudotuberculosis* IP32953) genomes. The genomes were mapped against the *Y*. *pestis* CO92 chromosome and plasmids without quality filter (-q 0, Samtools [samtools.sourceforge.net]. The resulting bam files were taken as input for Bedtools [69] to calculate the coverage of previously defined genes known to be involved in virulence. Based on these bed files a heat-map was generated to show their presence/absence (1 if the entire gene is present/covered; 0 if absent/no coverage) using the ggplot2 [70] package R Studio (v1.2.5033) [71] (S8 Fig).

## Supporting information

S1 Fig *In situ* molar pre (a) and post (b) removal of cementum, as well as pre (b) and post (c) sectioning and drilling of the pulp chamber and underlying dentin.
(TIF)

S2 Fig Superior Vertebral Arch Sampling. All sampling locations (post-drilling) of the thoracic vertebrae (superior view).
(TIF)

S3 Fig Femur (anterior view) showing drilling sites for the collection of both cortical and cancellous material. (TIF)

S4 Fig Melt curves for *pla* qPCR assay for KRA018 (left) and KRA028 and KRA032 (right) in pink, showing a peak at around 77°C. Standards are coloured in purple. A slight peak further to the right I blue (left figure) belonging to KRA023 was not considered positive due to its higher melting temperature.
(TIF)

S5 Fig Representative comparison of deamination patterns in reads from the KRA004 dental pulp chamber libraries mapping to both the HG19 human reference and *Yersinia pestis* CO92 reference genomes. (TIF)

S6 Fig Screenshots showing SNPs at positions (A) 699494, (B) 2262577, and (C) 1009185 for the low coverage genomes KRA008, KRA028, and KRA032. Reads shown here are untrimmed.
(TIF)

S7 Fig Unique SNPs at positions 1,009,185 (left) and 1,526,622 (right) of the KRA018 genome, covered by 26 and 4 reads, respectively.
(TIF)

S8 Fig Heatmaps showing read-based coverage of chromosomal (A) and plasmid-encoded (B) genes involved in virulence of *Y*. *pestis* for KRA004 and KRA018 (indicated in blue) and other ancient genomes as well as the CO92 reference genome and *Y*. *pseudotuberculosis* (IP32953). Coverage is given in %, where 100% denotes every nucleotide position in a gene covered with a minimum of 3-fold read support. Grey portions indicate regions where genetic absence is due to a lack of representation in the enrichment assay.
(TIF)

S1 Table EAGER mapping results for shotgun sequencing prior to *Y*. *pestis* capture. (XLSX)

S2 Table Results of *pla* qPCR assay performed in the course of the second, targeted screening. (XLSX)

S3 Table EAGER mapping to *Y*. *pestis* post capture. (XLSX)

S4 Table EAGER mapping of shotgun data against the human reference genome hg19. (XLSX)

S5 Table SNPs for ancient branch 1 genomes, limited to those that define diversity within the pestis secunda. (XLSX)

S6 Table Gene annotation and effects of SNPs that define diversity in the pestis secunda. (XLSX)
