# Supplementary figures and images for: 14th century Yersinia pestis genomes support emergence of pestis secunda within Europe
Source: PLoS Pathog. 2023 Jul 18;19(7):e1011404. doi: 10.1371/journal.ppat.1011404 (PMC10414589; doi:10.1371/journal.ppat.1011404)

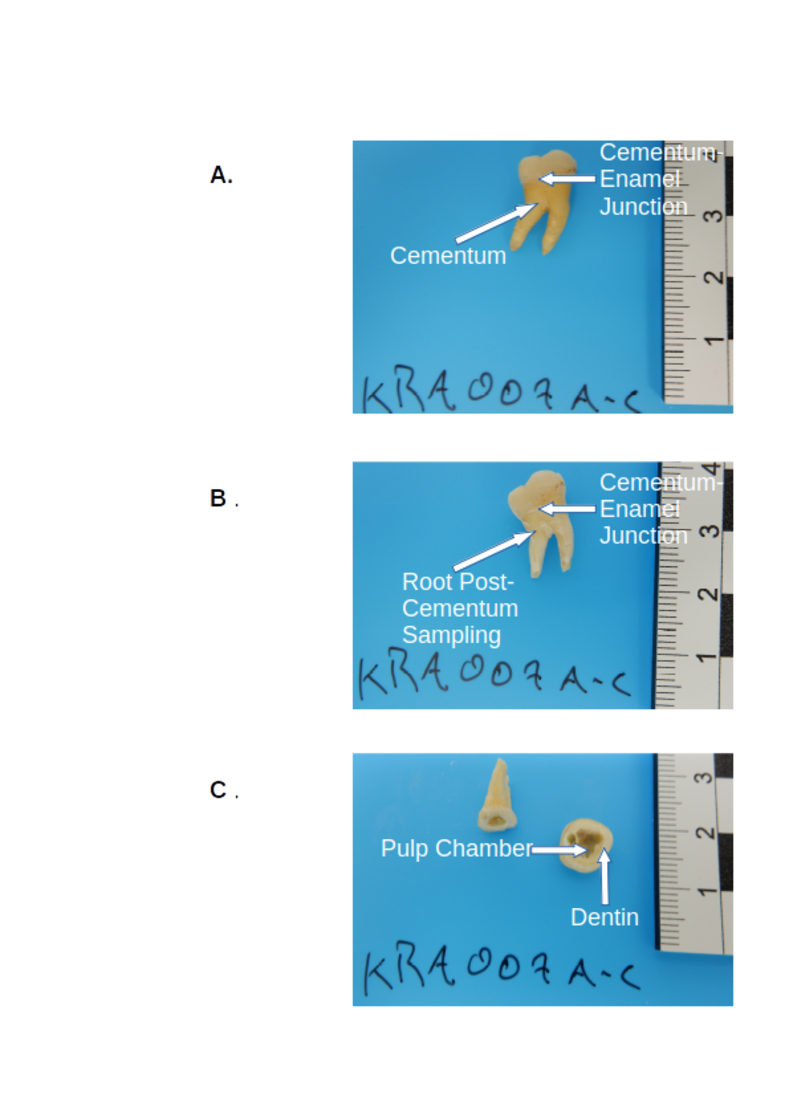

Supplement: S1 Fig — In situ molar pre (a) and post (b) removal of cementum, as well as pre (b) and post (c) sectioning and drilling of the pulp chamber and underlying dentin. (TIF) [file ppat.1011404.s001.tif]

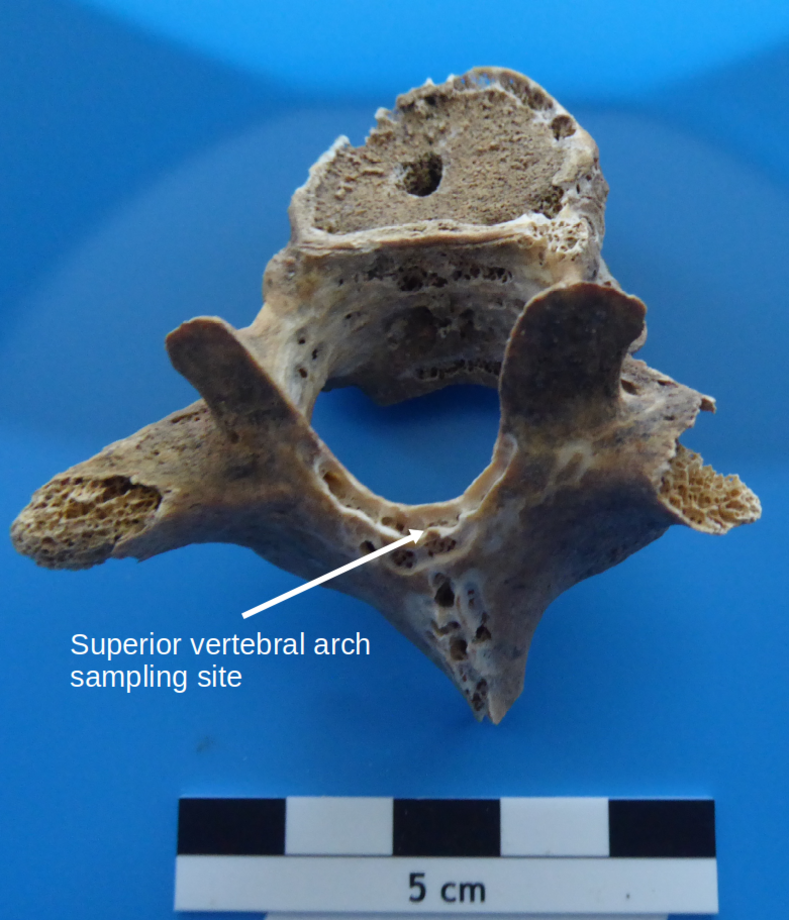

Supplement: S2 Fig — All sampling locations (post-drilling) of the thoracic vertebrae (superior view). (TIF) [file ppat.1011404.s002.tif]

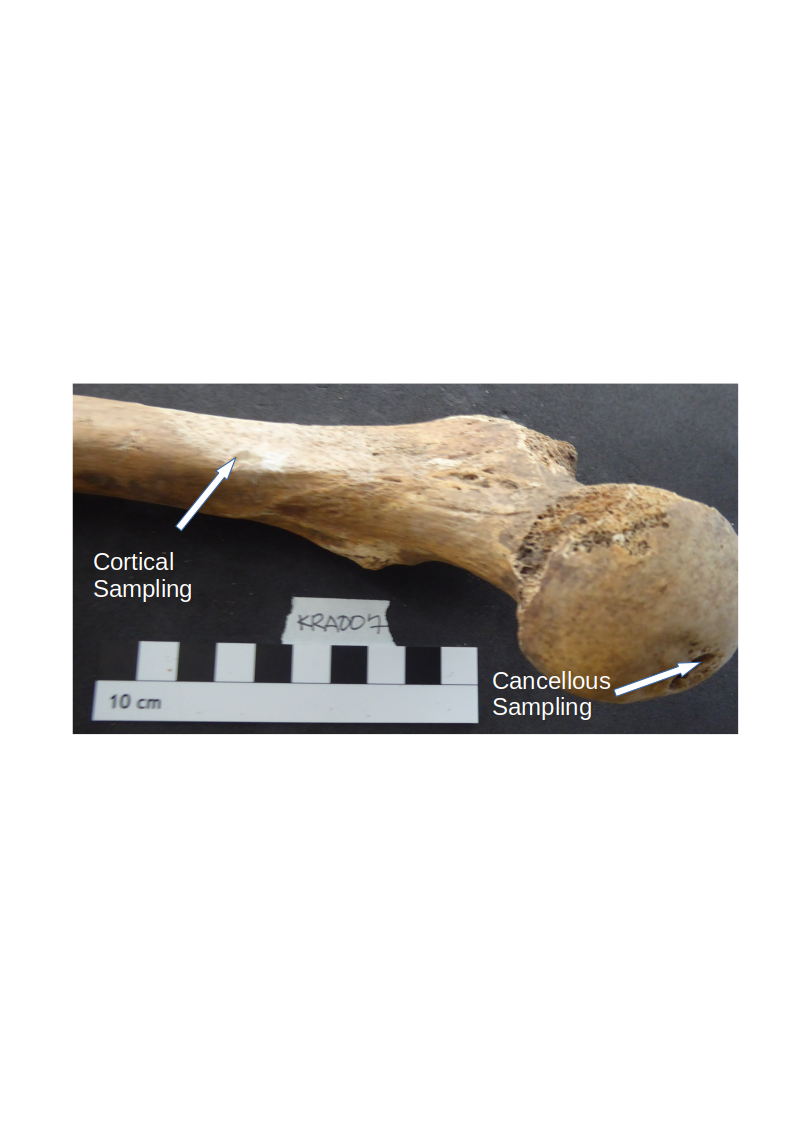

Supplement: S3 Fig — (TIF) [file ppat.1011404.s003.tif]

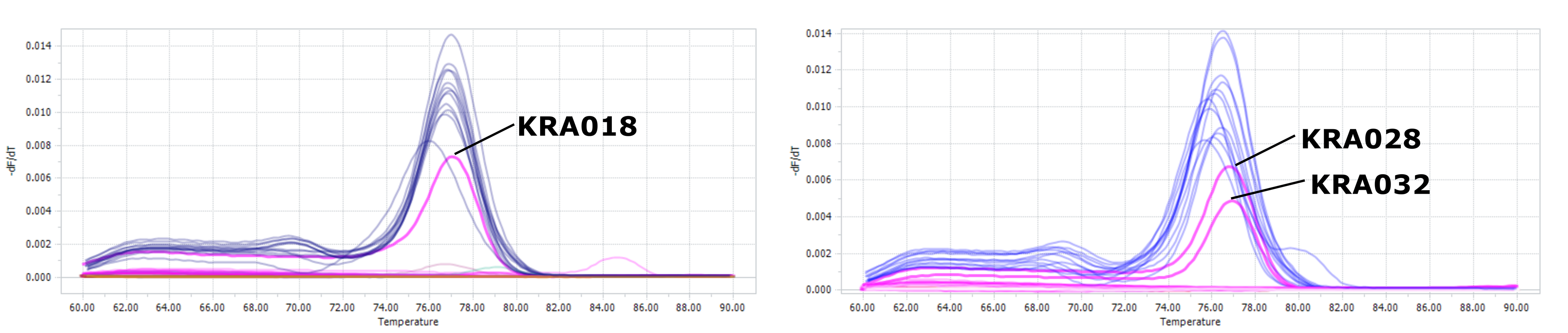

Supplement: S4 Fig — Melt curves for pla qPCR assay for KRA018 (left) and KRA028 and KRA032 (right) in pink, showing a peak at around 77°C. Standards are coloured in purple. A slight peak further to the right I blue (left figure) belonging to KRA023 was not considered positive due to its higher melting temperature. (TIF) [file ppat.1011404.s004.tif]

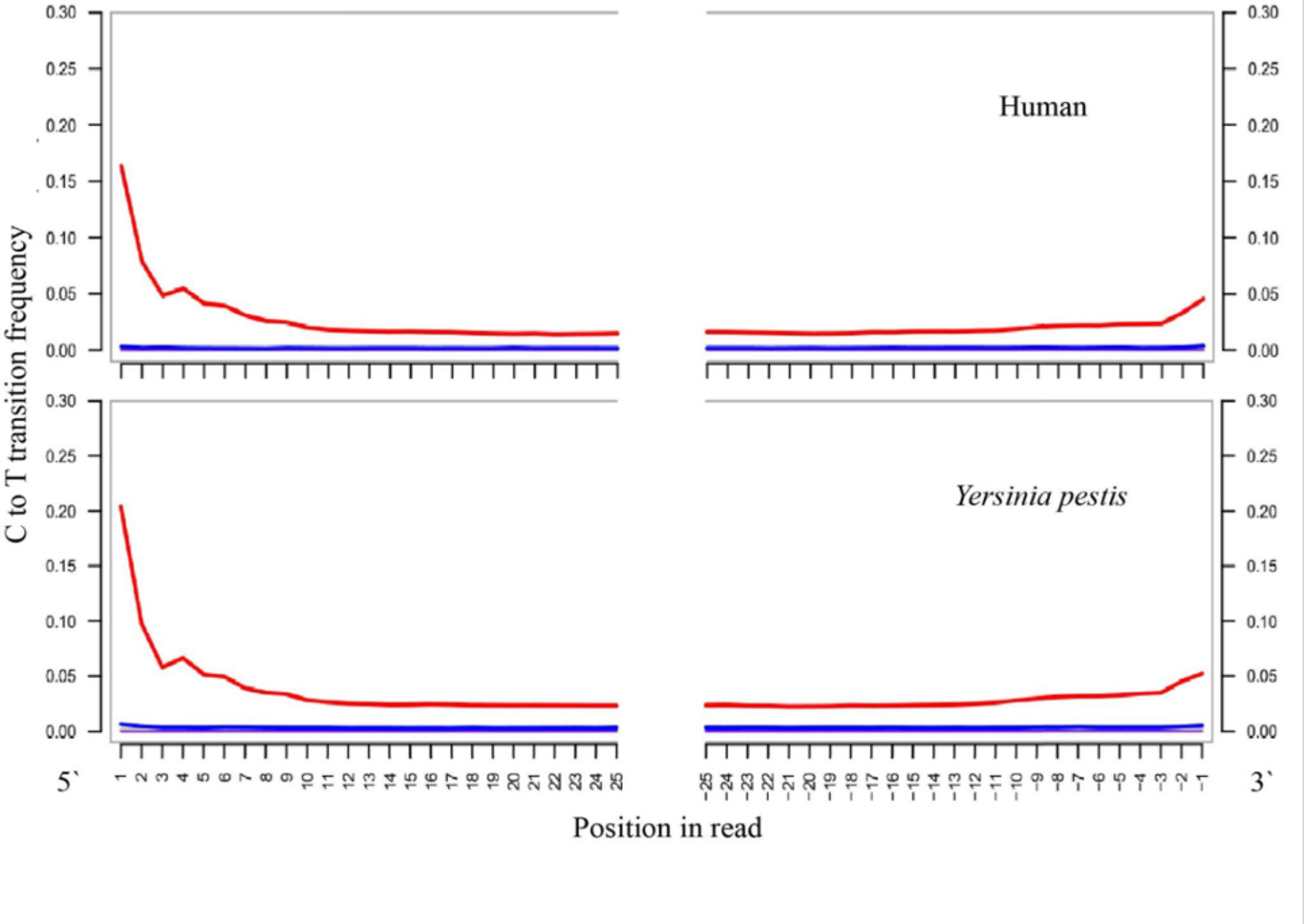

Supplement: S5 Fig — (TIF) [file ppat.1011404.s005.tif]

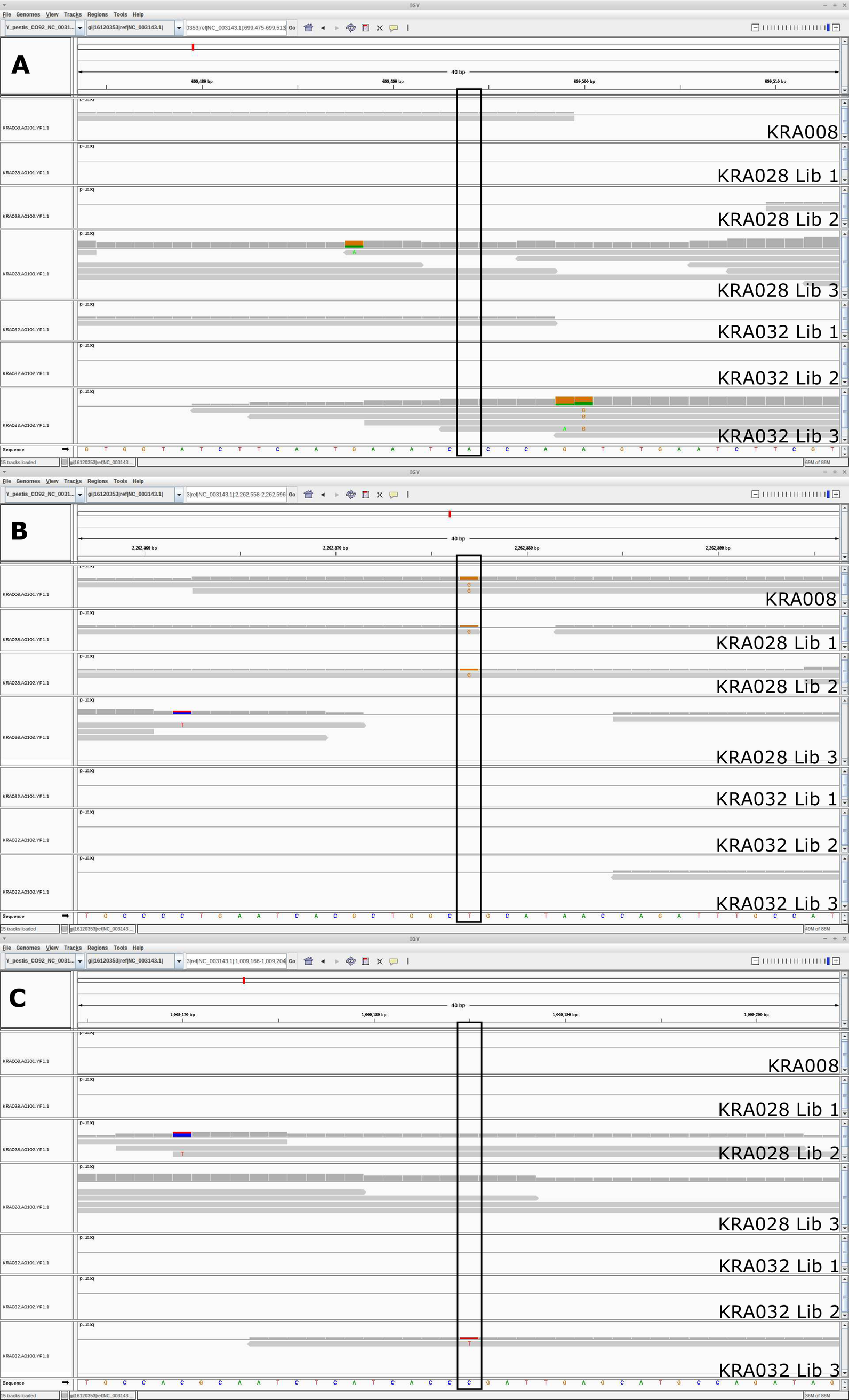

Supplement: S6 Fig — Screenshots showing SNPs at positions (A) 699494, (B) 2262577, and (C) 1009185 for the low coverage genomes KRA008, KRA028, and KRA032. Reads shown here are untrimmed. (TIF) [file ppat.1011404.s006.tif]

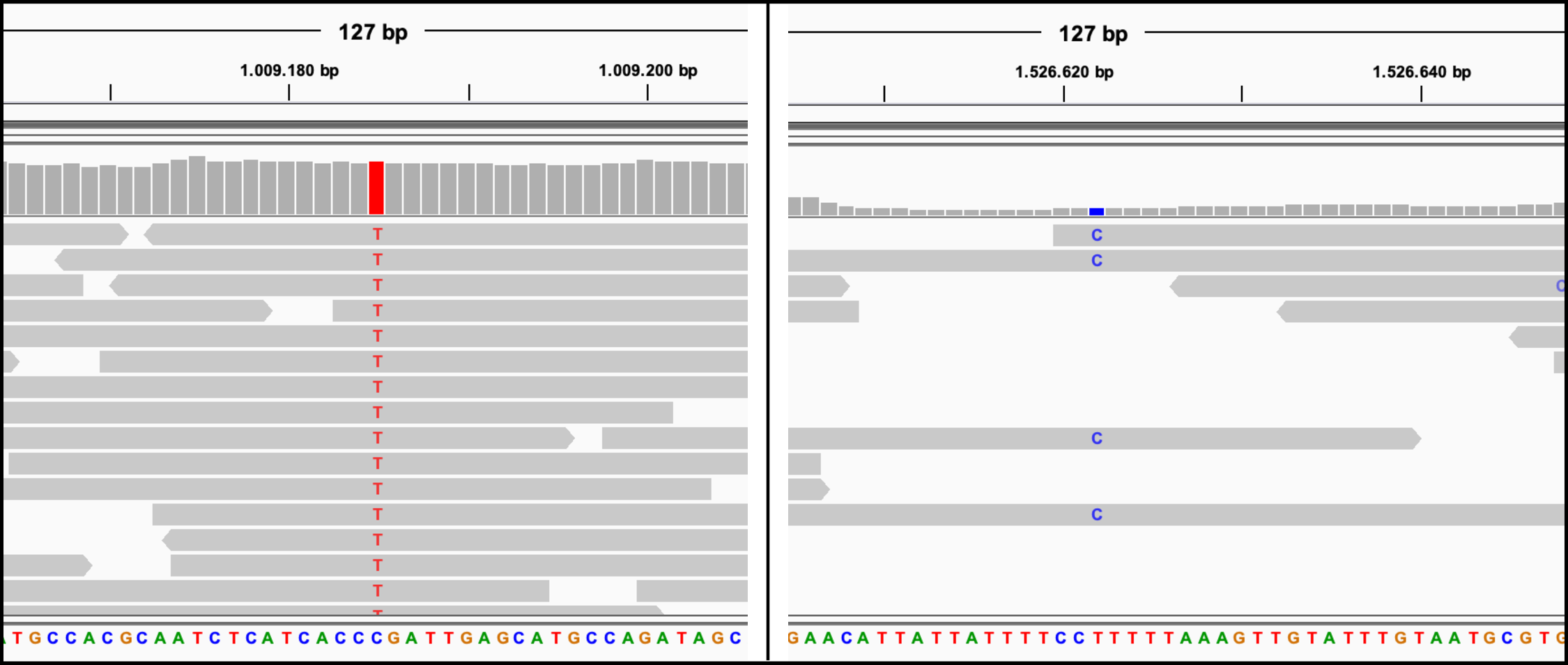

Supplement: S7 Fig — Unique SNPs at positions 1,009,185 (left) and 1,526,622 (right) of the KRA018 genome, covered by 26 and 4 reads, respectively. (TIF) [file ppat.1011404.s007.tif]

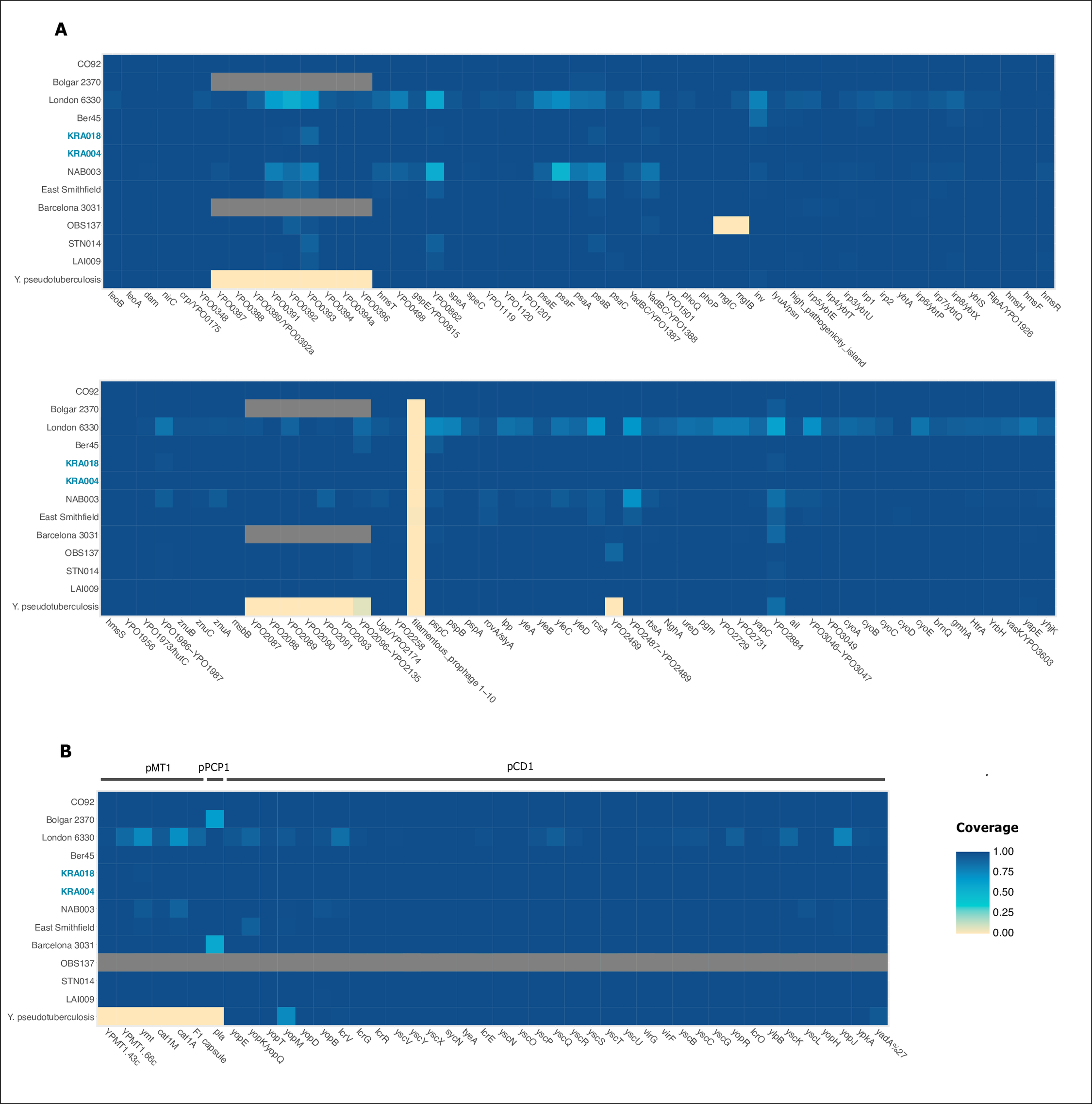

Supplement: S8 Fig — Heatmaps showing read-based coverage of chromosomal (A) and plasmid-encoded (B) genes involved in virulence of Y. pestis for KRA004 and KRA018 (indicated in blue) and other ancient genomes as well as the CO92 reference genome and Y. pseudotuberculosis (IP32953). Coverage is given in %, where 100% denotes every nucleotide position in a gene covered with a minimum of 3-fold read support. Grey portions indicate regions where genetic absence is due to a lack of representation in the enrichment assay. (TIF) [file ppat.1011404.s008.tif]
